# Clinical integration and application of the 2022 WHO pituitary tumor classification

**DOI:** 10.1093/noajnl/vdae145

**Published:** 2025-01-02

**Authors:** Sylvia L Asa, Shereen Ezzat, Ozgur Mete

**Affiliations:** Department of Pathology, University Hospitals Cleveland, Case Western Reserve University, Cleveland, Ohio, USA; Department of Medicine, Endocrine Oncology, University Health Network, University of Toronto, Toronto, Ontario, Canada; Department of Pathology, Endocrine Oncology, University Health Network, University of Toronto, Toronto, Ontario, Canada

**Keywords:** craniopharyngioma, pituitary blastoma, pituitary neuroendocrine tumors, pituitary pathology, pituitary tumors, WHO classification of pituitary tumors

## Abstract

The pituitary is a complex endocrine organ that gives rise to adenohypophysial epithelial neuroendocrine tumors, to hypothalamic neuroendocrine neuronal neoplasms, to nonendocrine glial-related pituicytomas, and to Rathke’s pouch-derived craniopharyngiomas and pituitary blastomas. The past 30 years have seen significant advances in the classification of these tumors based on cell lineages, molecular alterations, and biomarker features that distinguish tumor types and their subtypes. Adenohypophysial cells give rise to pituitary neuroendocrine tumors (PitNETs) that usually follow 1 of 3 cell differentiation pathways. Pituicytomas have multiple morphologic variants and hypothalamic neuronal tumors can be gangliocytomas composed of magnocellular neurons or neurocytomas composed of small neurocytes. Craniopharyngiomas have 2 distinct families, papillary and adamantinomatous, with different alterations in signaling pathways. Pituitary blastomas are primitive tumors composed of an admixture of immature elements of Rathke’s pouch with immature but differentiated hormone-secreting adenohypophysial cells. These various tumors are associated with specific clinical manifestations, creating a complex clinico-radiologic–pathologic classification that has both important clinical distinctions but also predictive therapeutic value. The syndromic disease has allowed the identification of genes that may be implicated in the development of these tumors, but the majority are sporadic tumors that, with a few exceptions, have no recurrent genetic alterations. The structure–function correlations that have been elucidated are critical to allow subclassification of clinical disorders that will pave the way for further advances in understanding genetic predisposition and environmental factors that alter epigenetic profiles of pituitary cells.

The pituitary is a major endocrine organ that is responsible for orchestrating growth, metabolic homeostasis, and reproduction.^1^ It does so by the production of at least 6 peptide hormones that are carefully regulated by central neural mechanisms as well as by feedback from target organ hormones in the circulation; the pituitary monitor’s stimulation and feedback suppression through complex signaling pathways. The gland is composed of 2 distinct portions; the anterior lobe and the posterior lobe. The anterior lobe, or adenohypophysis, is responsible for the production of the classical pituitary hormones including adrenocorticotropin (adrenocorticotropic hormone; ACTH), growth hormone (GH), prolactin (PRL), thyrotropin (thyroid stimulating hormone; TSH), and the gonadotropins follicle-stimulating hormone (FSH) and luteinizing hormone (LH). The posterior lobe, also known as the neurohypophysis, is an extension of axons of hypothalamic neurons embedded within modified glia known as pituicytes. The neurons produce a number of regulatory hormones that control adenohypophysial hormone production and also secrete oxytocin and vasopressin directly into the bloodstream for action or targets elsewhere. The adenohypophysis is delineated from the posterior lobe or neurohypophysis by remnants of Rathke’s cleft, the epithelial structure that arises during embryogenesis from the roof of the mouth; these structures are epithelial cysts with focal mucus production.

The adenohypophysial cells that produce hormones are epithelial neuroendocrine cells^2^ that are distributed throughout the anterior pituitary in a characteristic spatial distribution^1^; corticotrophs that produce ACTH are concentrated in the median wedge of the gland, lactotrophs that produce PRL are predominant in the posterolateral wings while somatotrophs that make GH and mammosomatotrophs that make both GH and PRL comprise about 80% of the lateral wings. Thyrotrophs that make TSH and gonadotrophs that make both FSH and LH are scattered throughout the gland.

Each component can give rise to tumors. *Adenohypophysial epithelial tumors*, formerly called “adenomas or pituitary adenomas” are now classified as pituitary neuroendocrine tumors (PitNETs) to reflect their derivation, spectrum of biological behavior, and response to therapies that are similar to neuroendocrine tumors at other body sites.^3^*Posterior lobe tumors* can be mature large cell gangliocytomas or smaller cell neurocytomas and pituicytes can give rise to the family of pituicytomas including the classical spindle cell tumors as well as oncocytic pituicytomas (formerly known as spindle cell oncocytomas) and granular pituicytomas (formerly called granular cell tumors) as well as the rare ependymal pituicytomas that resemble ependymomas.^4,5^*Remnants of Rathke’s cleft* give rise to Rathke’s cleft cysts and craniopharyngiomas.

The past 30 years have seen significant advances in the classification of these tumors based on cell lineages, molecular alterations, and biomarker features that distinguish tumor types and their subtypes.^6–8^

## Classification of Pituitary Tumors

Pituitary tumors are classified based on their cell differentiation.^6–8^ Tumors of adenohypophysial cells are ***PitNETs*** that are classified into 3 major families based on the expression of lineage-specific transcription factors and a fourth category is provided for those that either show no lineage differentiation or have promiscuous lineage infidelity (Table 1). The classification also notes the occurrence of multiple synchronous PitNETs that can be difficult to interpret but are evident on careful analysis.^9^ The term “pituitary carcinoma,” used previously to distinguish “pituitary adenomas” that developed metastases,^10^ is now reclassified as “***metastatic PitNETs***” in line with the WHO classification of neuroendocrine neoplasms.^11^ The term “neuroendocrine carcinoma,” reflecting a high-grade aggressive epithelial malignancy composed of poorly differentiated neuroendocrine cells, has yet to be formally ensconced in the pituitary literature as only a few cases have been reported.^12^

**Table 1.** Classification of Pituitary Neuroendocrine Tumors (PitNETs)

Transcription Factor Family	Cell Type	Tumor Type	Biomarkers
TPIT	Corticotroph	Densely granulated corticotroph tumor^b^	TPIT, PAS, ACTH, and keratins (diffuse cytoplasmic)
		Sparsely granulated corticotroph tumor^b^	TPIT, PAS (weak), ACTH (weak), and keratins (diffuse cytoplasmic)
		Crooke cell tumor^a^	TPIT, PAS, ACTH, and keratins (ring-like)
PIT1	Somatotroph	Densely granulated somatotroph tumor^b^	PIT1, GH (diffuse/strong), alpha-subunit, and keratins (perinuclear)
		Sparsely granulated somatotroph tumor^b^	PIT1, GH (weak), and keratins (fibrous bodies > 75%)
	Lactotroph	Densely granulated lactotroph tumor	PIT1, ER, and PRL
		Sparsely granulated lactotroph tumor	PIT1, ER, and PRL
	Mammosomatotroph	Mammosomatotroph tumor	PIT1, ER, GH (diffuse/strong), PRL, alpha-subunit, and keratins (perinuclear)
	Thyrotroph	Thyrotroph tumor	PIT1, GATA3, TSH, and alpha-subunit
	Mature plurihormonal PIT1-lineage cell	Mature plurihormonal PIT1-lineage tumor	PIT1, ER, GATA3, GH (diffuse/strong), PRL, TSH, alpha-subunit, and keratins (perinuclear)
	??	Immature PIT1-lineage tumor^a^	PIT1 ± ER ± GATA3 ± GH ± PRL ± TSH ± alpha-subunit
	??	Acidophil stem cell tumor	PIT1, ER, PRL, GH (weak), keratins (rare fibrous bodies), and alpha subunit (variable/focal)
SF1	Gonadotroph	Gonadotroph tumor	SF1, GATA3, FSH, LH, and alpha-subunit
None	Null cell	Null cell tumor^a^	None (a diagnosis of exclusion from other neuroendocrine neoplasms)
Multiple	??	Multilineage tumor	Multiple variables
	Multiple	Multiple synchronous PitNETs	Multiple variables but in distinct cell populations

Abbreviations: ACTH: adrenocorticotropic hormone; ER: estrogen receptor; FSH: follicle-stimulating hormone; GH: growth hormone; LH: Luteinizing hormone; PAS: periodic acid Shiff; PIT1: pituitary transcription factor 1; PRL: prolactin; SF1: steroidogenic factor 1; TPIT: T-box transcription factor; TSH: thyroid stimulating hormone.

^a^These tumors are thought to be more aggressive.

^b^Sparsely granulated tumors tend to be larger and more invasive with less florid features of hormone excess than densely granulated counterparts.

***Craniopharyngiomas*** are divided into adamantinomatous and papillary craniopharyngiomas based on their distinct morphologies and pathogenesis; the former have altered WNT signaling due to mutations that result in nuclear translocation of beta-catenin; the latter harbor activation mutations of *BRAF*, most frequently resulting in expression of *BRAF* p.V600E that can be detected by mutation-specific VE1 immunohistochemistry.^13,14^

The rare ***pituitary blastoma*** is a primitive tumor composed of immature elements of Rathke’s pouch along with some immature but terminally differentiated adenohypophysial cells; these neoplasms of early childhood are almost always reflective of pathogenic germline *DICER1* variants^15–17^ but young adult manifestations have also been described.^18^

Tumors of the posterior lobe and hypothalamus include the various subtypes of TTF1-expressing pituicyte-lineage tumors (***pituicytomas***) as mentioned above, as well as hormone-producing ***gangliocytomas and sellar neurocytomas***.^19^ Gangliocytomas are often composite tumors that may be associated with a PitNET. Neurocytomas may be associated with vasopressin excess.^20^

## Clinical Correlates

Pituitary tumors may be associated with a number of clinical syndromes due to hormone excess that can be florid or subtle (Table 2). The majority of tumors are hormonally silent clinically and biochemically but hormone expression in tumor tissue, along with transcription factor expression, allows accurate classification that has prognostic and predictive value.^6,8^

**Table 2.** Clinico-pathologic Correlations of Pituitary Neuroendocrine Tumors (PitNETs)

Clinical Presentation	Tumor type	Clinical and Radiologic Features
Hyperprolactinemia (Galactorrhea-Amenorrhea)^b^	Sparsely granulated lactotroph tumor	Prolactin levels typically correlate with tumor size; respond to dopamine agonist therapy
	Densely granulated lactotroph tumor	Usually large and less likely to respond to dopamine agonist therapy
	Acidophil stem cell tumor	Predominant hyperprolactinemia with “fugitive” acromegaly
	^a^Immature PIT1-lineage tumor	Hyperprolactinemia ± acromegaly ± hyperthyroidism, large infiltrative tumor
	Mammosomatotroph tumor	Florid acromegaly + hyperprolactinemia, small tumor, hypo-/isointense on T2 MRI
	^a^Mature plurihormonal PIT1-lineage tumor	Florid acromegaly + hyperprolactinemia + hyperthyroidism, small tumor, and hyper-/isointense T2 weighted MRI
Cushing disease	Densely granulated corticotroph tumor	Florid Cushing, small tumor, hypo-intense on T2 MRI
	Sparsely granulated corticotroph tumor	Subtle Cushing, large tumor, hyper-intense T2 MRI
	Crooke cell tumor	Variable Cushing, large tumor
GH/IGF1 excess (acromegaly/gigantism)	Densely granulated somatotroph tumor	Florid acromegaly, small tumor, hypo/isointense T2 MRI
	Sparsely granulated somatotroph tumor	Subtle acromegaly or early onset, large tumor, hyper-intense on T2 MRI
	Mammosomatotroph tumor	Florid acromegaly + hyperprolactinemia, small tumor, hypo-/isointense on T2 MRI
	^a^Mature plurihormonal PIT1-lineage tumor	Florid acromegaly + hyperprolactinemia + hyperthyroidism, small tumor
	^a^Immature PIT1-lineage tumor	Variable acromegaly ± hyperprolactinemia ± hyperthyroidism, large infiltrative tumor
	Acidophil stem cell tumor	Predominant hyperprolactinemia with “fugitive” acromegaly
Hyperthyroidism	Thyrotroph tumor	Hyperthyroidism
	^a^Mature plurihormonal PIT1-lineage tumor	Hyperthyroidism with GH excess, usually small tumor
	^*^Immature PIT1-lineage tumor	Hyperthyroidism ± hyperprolactinemia ± GH excess, large infiltrative tumor
Mass	Any PitNET or non-PitNET, inflammatory lesion	
Multiple	Multiple synchronous PitNETs	
	Multilineage PitNET	

^a^Note that the same tumor type may present with different clinical manifestations.

^b^Variable PRL < 150 ug/L (mIU/L) may occur with any other lesion with stalk interruption.

Abbreviations: GH: growth hormone; IGF1: insulin-like growth factor 1; MRI: magnetic resonance imaging.

PitNETs are the most common tumor; while they occur in up to 20% of the population, they are often not detected clinically and may be found as incidental lesions on imaging or at autopsy.^1^ They have a prevalence of 78–116 cases per 100 000 people and an estimated incidence of 4.12/100 000 population per year.^1^ Prolactin-producing tumors are the most common functioning tumors and most are treated medically; their true incidence is not captured in cancer statistics. Among surgically resected PitNETs, about 40% are clinically silent and the vast majority of those are gonadotroph tumors; PIT1-lineage tumors represent about 30% and half of those are associated with GH excess while ACTH-producing tumors represent about 15%.^21^ Craniopharyngiomas constitute less than 5% of surgically managed intracranial tumors while other primary sellar pituitary and hypothalamic tumors are relatively rare.

Prolactin excess results in a syndrome that is more often obvious in younger women due to amenorrhea and galactorrhea but can be subtle, especially in older patients who manifest loss of libido, and osteoporosis. Hyperprolactinemia in a patient with a pituitary mass can be the result of tumor production but also occurs in patients with masses that do not produce PRL but interrupt the tonic hypothalamic inhibition of PRL secretion by dopamine, including even inflammatory disorders. In patients with clear tumor masses, which are often dismissed clinically as “PRLomas,” it is critically important for the diagnostician to analyze PRL levels in the context of tumor size. In general, large tumors that do not produce PRL result in PRL levels that do not exceed 150 ug/L (mIU/L, where normal is <40 in nonpregnant females). Among tumors that produce PRL, the vast majority are sparsely granulated lactotroph tumors that show a very strong correlation between tumor size and degree of hyperprolactinemia. These highly differentiated PitNETs are exquisitely susceptible to dopamine agonist therapies that result in tumor shrinkage and normalization of PRL levels,^22^ and over the past 40 years the availability of novel dopamine agonists has resulted in a significant reduction of patients requiring surgery and some ultimately resolve with medical therapy alone. Those that come to surgery are either very large “giant” tumors or other tumor types that produce PRL, including the rare densely granulated lactotroph tumors, acidophil stem cell tumors and immature PIT1-lineage tumors (formerly known as silent subtype 3 pituitary adenomas or poorly differentiated PIT1-lineage tumors^6,23^). Hyperprolactinemia is also a feature of mammosomatotroph and mature plurihormonal PIT1-lineage tumors but these are almost always associated with GH excess that dominates the clinical scenario.^1,6^

Acromegaly and/or gigantism are the result of GH excess; the former is more common and is an adult-onset disease whereas the latter results from GH excess prior to epiphyseal fusion at puberty. Acromegaly has traditionally been considered a single clinical disease but it is now clear that it results from many different tumor types^24,25^ and each has a distinct clinical presentation, prognosis and therapeutic approach. The most common cause is a densely granulated somatotroph tumor that results in florid clinical features, has its onset usually around the age of 50 years, and is hypointense of T2-weighted magnetic resonance imaging (MRI).^1,24–26^ Because of the florid features, these lesions are often intrasellar at the time of diagnosis, but they may be invasive and not surgically resectable. They are usually sensitive to somatostatin analogue therapy that can be used preoperatively to facilitate the surgical approach by shrinking soft tissue, and can also be used for postoperative treatment in patients with unresectable disease.^27,28^ In contrast, the sparsely granulated somatotroph tumors that release less GH cause more subtle clinical features but have more aggressive growth, and the average age of onset is 10 years younger than in patients with densely granulated somatotroph tumors. Sparsely granulated somatotroph tumors are hyperintense of T2-weight MRI and tend to be widely invasive at the time of diagnosis^24–26^; they are less responsive to first-generation somatostatin analogs.^24,25,28^ Mammosomatotroph tumors and mature plurihormonal PIT1-lineage tumors are clinically and radiologically similar to densely granulated somatotroph tumors but the former also secrete PRL and the latter may secrete TSH as well as PRL, resulting in more complex clinical scenarios.^1,6^ Immature PIT1-lineage tumors are more aggressive neoplasms that may cause acromegaly in 10–20% of patients and may occur in younger patients.^23^ The unusual acidophil stem cell tumor may cause a fluctuating syndrome of GH and IGF-1 excess with extremely subtle or nonexistent clinical features; this “fugitive acromegaly”^29^ remains a significant clinical challenge. Interestingly the risk of altered glucose metabolism is also impacted by the tumor type causing acromegaly.^30^ In exceptional cases GH excess may be caused by a hypothalamic GHRH-producing gangliocytoma with or without an associated PitNET, or by ectopic GHRH or GH production by a neuroendocrine neoplasm elsewhere in the body.

TSH excess has traditionally been attributed to tumors called “TSHomas” however this has resulted in very confusing literature since this group of PitNETs includes both pure thyrotroph tumors as well as mature plurihormonal and immature PIT1-lineage tumors.^31^ Some literature has claimed that these are aggressively infiltrative and fibrotic tumors but that is true only of immature PIT1-lineage tumors.^23^ The incidence of a thyrotroph tumor is largely unknown in most series; however, its incidence is 0.07% in a large series characterized by rigid criteria that are the standards of practice as per 2022 WHO classification.^21^

Cushing disease, like acromegaly, is actually attributable to a spectrum of pituitary pathologies including the classic densely granulated corticotroph tumor that causes florid clinical features and tends to be small and hypointense on T2-weighted MRI, and the sparsely granulated corticotroph tumor that can give rise to only borderline clinical and biochemical features and tends to be larger and more invasive with hyperintense T2-weighted MRI features^1,6,32^; the latter has in some publications been classified as a “silent” corticotroph tumor but this is incorrect. Truly “silent” tumors have no clinical or biochemical features of hormone production. The interpretation is based on the acuity of the clinician(s) who need to ensure that they are aware of the subtle features of cortisol excess and how to establish the diagnosis using appropriate biochemical assessment. An unusual tumor that can cause cyclical or atypical Cushing disease is the Crooke cell tumor. Crooke’s hyaline change is an accumulation of hyaline keratin filaments in nontumorus corticotrophs that are subjected to cortisol excess, endogenous or exogenous; it is a feature of the nontumorous gland in patients with functioning corticotroph tumors.^33^ As an example of complete dissociation of hormone regulatory function and proliferation, tumors composed of corticotrophs with Crooke’s hyaline change are highly aggressive neoplasms that may cause ACTH and cortisol excess.^34^ As with acromegaly, the differential diagnosis includes ectopic sources of ACTH and/or CRH,^35^ hypothalamic CRH or vasopressin production by tumors^20,36^ and some patients have an idiopathic disorder known as primary corticotroph “hyperplasia.”^37^ Unusual causes in childhood also include pituitary blastoma.^38^

Hormonally inactive tumors, clinically known as “clinically nonfunctioning” tumors, present with signs and symptoms of a sellar mass such as visual disturbances, headache, and/or nausea, and evidence of hypopituitarism. These tumors are most often composed of gonadotrophs^21^ but may be null cell tumors that express no hormones or lineage-specific transcription factors,^39,40^ or they may be silent differentiated PitNETs, the most common being truly silent corticotroph tumors of either the densely or the sparsely granulated subtype.^41^ Functioning gonadotroph tumors are exceptionally rare. Gonadotropin (FSH and LH)-negative gonadotroph tumors are more common in older patients, and the status of gonadotropin expression does not appear to correlate with tumor invasiveness or tumor proliferative activity.^42^

Other lesions fall into this clinical category including pituicytomas, neurocytomas and gangliocytomas as well as craniopharyngiomas, and the possibility of unusual primary tumors (such as olfactory neuroblastoma or paraganglioma), stromal or germ cell tumors, inflammatory lesions^43^, and metastatic disease must always be considered.^1^ The clinical and imaging characteristics should prompt consideration of these other diagnoses. Adenohypophysial cell lineage-specific markers such as SF1, TPIT, and PIT1 should be examined in the workup of a nonfunctional sellar tumor with neuroendocrine differentiation,^7^ and the relevance of GATA3 must also be recognized as a nonspecific marker in this differential diagnosis.^44^

## Molecular Genetics and Syndromic Disease

The majority of PitNETs are sporadic tumors but some are familial and the mutations underlying the familial and syndromic tumors have shed light on the pathogenesis of these neoplasms.^16,45,46^ Some tumors are manifestations of multiple endocrine neoplasia (MEN) type 1 or, more rarely type 4 or 5 syndrome.^47^ Sparsely granulated somatotroph tumors are associated with the isolated familial somatotropinoma syndrome (also known as familial isolated pituitary adenoma or FIPA syndrome) that is due to mutations in *AIP*.^16,17^ Occasional patients have *SDH*x disease^48^ and rare tumors have been seen in a patient with HPTJT syndrome due to mutation of *CDC73*.^49^ Patients with McCune-Albright syndrome and those with Carney complex develop densely granulated somatotroph or mammosomatotroph PitNETs.^16,17^ Early childhood-onset disease is frequently associated with DICER1 syndrome^15^ or X-linked acrogigantism (X-LAG, due to Xq26 microduplication or GPR101 alterations).^50,51^ Patients with Lynch syndrome are known to develop PitNETs^52^ and other less well-known genetic variants may predispose to the development of these tumors^53^ and may be implicated in altering hormone secretion.^53–57^

In the case of sporadic tumors, the majority do not have recurrent genetic alterations and the disease is thought to be attributed to epigenetic alterations that may be associated with environmental challenges or stress.^58^ A few specific tumor types have a clear pattern of genetic changes that have predictive value: the densely granulated somatotroph tumors have activating *GNAS* mutations that provide the mechanism for their somatostatin-responsiveness, and densely granulated corticotroph tumor have been reported to harbor mutations of *USP8, USP48* or *BRAF.*

In the case of craniopharyngiomas, the identification of *BRAF* p.V600E expression predicts response to medical therapy with BRAF inhibitors.^13^

## Future Directions

The alignment of pituitary-derived NETs with other body sites is undoubtedly bringing the topic to the forefront of discussion and consideration. While some may argue that these are not neoplasms deserving of tumor assignment, such concerns dissipate when considering earlier clinical experiences. For instance, the pituitary is a component of MEN syndromes that have superseded the older multiple endocrine adenomas syndrome. Regardless of adoption, the PitNET classification is expected to harness the translational knowledge and logistical derivatives of advances in the broader field of NETs. This will likely include the refinement of molecular diagnostic, imaging, and therapeutic strategies.
